# Amelioration of Lipopolysaccharide-Induced Acute Lung Injury in Rats by Na-H Exchanger-1 Inhibitor Amiloride Is Associated with Reversal of ERK Mitogen-Activated Protein Kinase

**DOI:** 10.1155/2018/3560234

**Published:** 2018-12-09

**Authors:** Yan Zhang, Hao He, Boran Zhang, Qinghong Chen, Shanglong Yao, Ping Gui

**Affiliations:** ^1^Department of Anesthesiology, Union Hospital, Tongji Medical College, Huazhong University of Science and Technology, Wuhan, China; ^2^Medical College of Hubei Polytechnic University, Huangshi, China

## Abstract

**Background:**

Na-H exchanger-1 (NHE-1) is expressed in the lung of rats. Accumulating evidence shows that Na-H exchangers are involved in inflammation. Amiloride, an inhibitor of NHE-1, inhibits the activation of macrophages and endothelial cells and reduces their production of cytokines. Since these processes have been implicated in acute lung injury (ALI) induced by lipopolysaccharide (LPS), we examined the protective effect of amiloride on ALI induced by LPS in rats.

**Material and Methods:**

ALI in specific pathogen-free male Sprague-Dawley rats was induced by an intravenous injection of 6 mg/kg LPS. Amiloride pretreated rats received an intravenous injection of 10 mg/kg amiloride 30 min before the administration of LPS. Controls received normal saline in a similar manner. All animals were sacrificed 6 h after LPS or normal saline administration. The degree of ALI was assessed by wet-to-dry weight ratio (W/D) and lung histological examination. Neutrophilic infiltration was determined by myeloperoxidase (MPO) activity in lung tissue. Concentrations of total protein (TP), tumor necrosis factor-alpha (TNF-*α*), and macrophage inflammatory protein-2 (MIP-2) in bronchoalveolar lavage fluid (BALF) were also measured. Expression of NHE-1 and mitogen-activated protein kinase (MAPK) p38, p-p38, ERK, and p-ERK was evaluated by western blot analysis.

**Results:**

Pretreatment with amiloride significantly reduced the increase in W/D, ALI score, lung tissue MPO activity, concentrations of TP, TNF-*α*, and MIP-2 in BALF, resulting in attenuation of ALI induced by LPS. Meanwhile, levels of NHE-1 and p-ERK proteins were reversed, whereas that of p-p38 was not.

**Conclusions:**

These findings suggest that NHE-1 inhibitor amiloride could attenuate ALI induced by LPS in rats. This effect is mediated through reversal of ERK.

## 1. Introduction

Lipopolysaccharide (LPS), a gram-negative bacterial outer membrane component, causes multiple organ dysfunction, including acute lung injury (ALI) or its more severe form acute respiratory distress syndrome (ARDS) [1]. They are a major cause of mortality in intensive care units. The pathogenesis of ALI is not well understood, but damage of the lung epithelium and endothelium, the expression of proinflammatory mediators, and an extensive neutrophil influx into the lungs are thought to play an important role [2]. 

Na-H exchangers (NHEs) are transmembrane proteins that exchange one intracellular H^+^ for one extracellular Na^+^ and play a role in the regulation of intracellular pH, cell volume, vectorial ion transport, and cell proliferation. To date, nine isoforms (NHE-1-NHE-9) have been identified and cloned. These isoforms differ in their distribution, regulatory properties, and physiological functions [3, 4]. Lungs have been found to express NHE-1 [5].

NHEs are activated by a variety of inflammatory stimuli, including LPS, interleukin-1 (IL-1), tumor necrosis factor-alpha (TNF-*α*), and interferon-*γ* (IFN-*γ*) [6–9]. NHEs have been implicated in the regulation of inflammatory mediator production. Inhibition of activated NHEs suppresses IL-8 production in LPS-stimulated macrophages, endothelial cells, and intestinal epithelial cells [10–12]. Inhibition of activated NHEs also suppresses prostaglandin E2 (PGE2), macrophage inflammation protein-1*α* (MIP-1*α*), and macrophage inflammatory protein-2 (MIP-2) production in LPS-stimulated macrophages [13, 14]. There is accumulating evidence that the activation of NHEs contributes to endothelial cell injury during many pathophysiological processes associated with acidosis and hypoxia [15]. Inhibition of NHEs prevents burn-induced multiple organ injury or improves tissue perfusion and resuscitation outcome after severe hemorrhage [16, 17]. Because all of these processes have been implicated in ALI induced by LPS, we investigated whether NHE-1 inhibitor amiloride would attenuate ALI induced by LPS in rats and its associated mechanisms.

## 2. Materials and Methods

### 2.1. Animals

This study was conducted according to the guidelines of animal study in China, and the study protocol was approved by the Laboratory Animal Review Board of Tongji Medical College. Specific pathogen-free male Sprague-Dawley rats weighing 200-250 g were purchased from the Laboratory Animal Center of Tongji Medical College. Rats were housed in a pathogen-free laboratory with free access to water and food.

### 2.2. Experimental Protocol

The animals were randomly divided into four groups and anesthetized by intraperitoneal injection of 20% urethane (1.5g/kg). Following anesthesia, a 24-gauge cannula was inserted into the jugular vein for the administration of fluid and drugs. Both amiloride (Sigma, USA) and LPS (*E. Coli* O55:B5, Sigma, USA) were dissolved in normal saline. The control group (C group, n=8) received an intravenous injection of normal saline, the amiloride group (A group, n=8) received an intravenous injection of 10 mg/kg amiloride, the LPS group (L group, n=8) received an intravenous injection of 6 mg/kg LPS, and the amiloride and LPS group (AL group, n=8) received an intravenous injection of 10 mg/kg amiloride 30 min before LPS administration. All animals were sacrificed by exsanguination 6 h after LPS or normal saline administration.

### 2.3. Preparation of Bronchoalveolar Lavage Fluid (BALF)

The chest was opened and the right principal bronchus was ligated immediately after animals were sacrificed. Bronchoalveolar lavage was performed with three 2 ml aliquots of 4°C normal saline injected into the trachea and gently withdrawn. The recovered fluid was centrifuged at 1200 g at 4°C for 10 min, and supernatants were divided into aliquots and stored at -70°C for future assay of protein and cytokines.

### 2.4. Wet-to-Dry Weight Ratio

The severity of pulmonary edema was assessed using the wet-to-dry weight ratio (W/D) of the lung. The right upper lobe was excised, weighed in a tared container and then dried in a drying oven at 70°C until a constant weight was obtained, and the W/D was calculated.

### 2.5. Histopathologic Evaluation

The right lower lobe was excised. Approximately 100 mg lung tissue was prepared for western blot analysis. The right lower lobe left was fixed by immersion in 4% paraformaldehyde, dehydrated with a graded alcohol series, and embedded in paraffin. Paraffin-embedded sections were stained with hematoxylin and eosin and scored by a pathologist who was blinded to the protocol and experimental groups. Lung injury was scored according to the following four items: (1) alveolar congestion, (2) hemorrhage, (3) infiltration or aggregation of neutrophils in the airspace or vessel wall, and (4) thickness of the alveolar wall/hyaline membrane formation [18]. Each item was graded according to the following five-point scale: 0 = minimal damage; 1 = mild damage; 2 = moderate damage; 3 = severe damage; and 4 = maximal damage. A total score of 0 indicated normal histology, and that of 16 indicated maximal damage.

### 2.6. Measurement of Total Protein and Cytokines

Total protein in BALF was determined using a commercialized BCA Protein Assay Kit (Pierce, USA) according to the manufacturer's protocol. Concentrations of TNF-*α* and MIP-2 in BALF were also measured by using commercially available ELISA kits (Biosource, USA) according to the manufacturer's protocol.

### 2.7. Myeloperoxidase (MPO) Activity Assay

As an index of neutrophil infiltration, tissue-associated MPO activity was assessed by using a commercial kit (Nanjing Jiancheng Bioengineering Institute, Nanjing, China) according to the manufacturer's protocol. The results were expressed as units per gram of wet tissue.

### 2.8. Protein Extraction and Western Blot Analysis

Briefly, approximately 100mg lung tissue samples were homogenized in 0.5 ml of ice-cold buffer, composed of 10.0 mM HEPES (pH7.9), 10.0 mM KCl, 0.1 mM EDTA, 2 mM MgCl_2_, 1.0 mM dithiothreitol, and 0.5 mM phenylmethylsulfonyl fluoride. The homogenates were centrifuged at 450×g at 4°C for 1 min. The supernatants were collected and incubated on ice for 15 min, vortexed for 30 s after the addition of 50 *μ*L 10% NP-40, and then centrifuged at 6 000×g at 4°C for 1 min. The supernatants were collected as cytosol extracts and stored at -70°C. Protein concentration was determined by a Bradford-based assay [19].

Fifty micrograms of protein was electrophoresed in a 10% SDS-PAGE and transferred to a nitrocellulose membrane. The membrane was blocked for 1 h at room temperature with blocking solution (5% nonfat milk in Tris buffered saline with Tween 20). Blots were then incubated with primary antibodies against NHE-1 (Chemicon, USA), p38, p-p38, ERK, or p-ERK (Cell signaling, USA) overnight at 4°C. Then, the membrane was washed in 5% nonfat in Tris buffered saline with Tween 20 and was incubated with a horseradish peroxidase-labeled secondary antibody for 1 h at room temperature. Immunoreactive proteins were visualized using enhanced chemiluminescence detection (Amersham Biosciences, USA) and exposed to radiograph film. The autoradiograph was assessed semiquantitatively using computer-assisted densitometry.

### 2.9. Statistical Analysis

Data were expressed as mean ± SD. The statistical analysis was performed using SPSS 10 software (SPSS Inc., Chicago, IL). All data were analyzed by Student's* t *test or one-way analysis of variance, and a difference with a* p*<0.05 was considered statistically significant.

## 3. Results

### 3.1. Wet-to-Dry Weight Ratio

**Figure 1:**
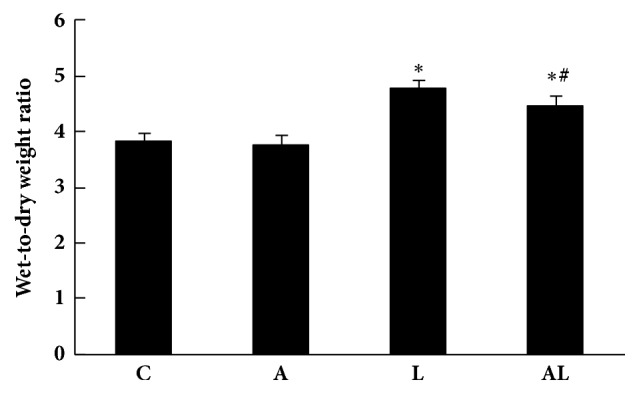
Lung wet-to-dry ratios of groups. Values are mean ± SD (n=8 per group). ^∗^*p*<0.01 versus C group; ^#^*p*<0.01 versus L group.

The W/D increased significantly in the L group compared with the C group (*p*<0.01, Figure 1). This increase was significantly reduced in the AL group (*p*<0.01). However, it was significantly higher than that of the C group (*p*<0.01).

### 3.2. Histopathologic Evaluation

**Figure 2:**
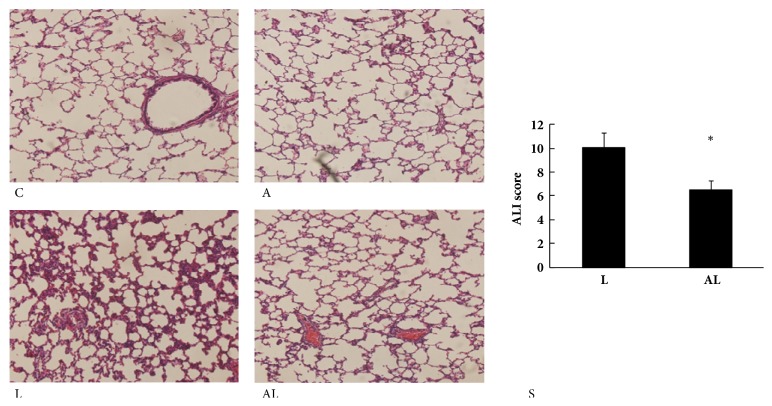
Morphologic changes and acute lung injury scores of lungs. (C) C group: no lung injury was observed. (A) A group: no lung injury was observed. (L) L group: the inflammatory process with marked infiltration of leukocytes into interstitial and alveolar spaces, edema, alveolar distortion, and thickening of alveolar wall. (AL) AL group: the damage was less pronounced. Hematoxylin-eosin-safranin stain was applied to the sections. Original magnification, 200X. (S) Acute lung injury scores of groups. Values are mean ± SD (n=8 per group). ^∗^*p*<0.05 versus L group.

The C group and the A group showed normal pulmonary histology (Figures 2(C) and 2(A)), whereas the lung tissues from the L group were significantly damaged, with interstitial edema, thickening of the alveolar wall, and infiltration of inflammatory cells into the alveolar and interstitial spaces (Figure 2(L)). These morphologic changes were less pronounced in the AL group (Figure 2(AL)). The lung injury score of the AL group was significantly lower than that of the L group (*p*<0.05, Figure 2(S)).

### 3.3. Protein, Cytokines in BALF and MPO Activity in Lung Tissue

**Figure 3:**
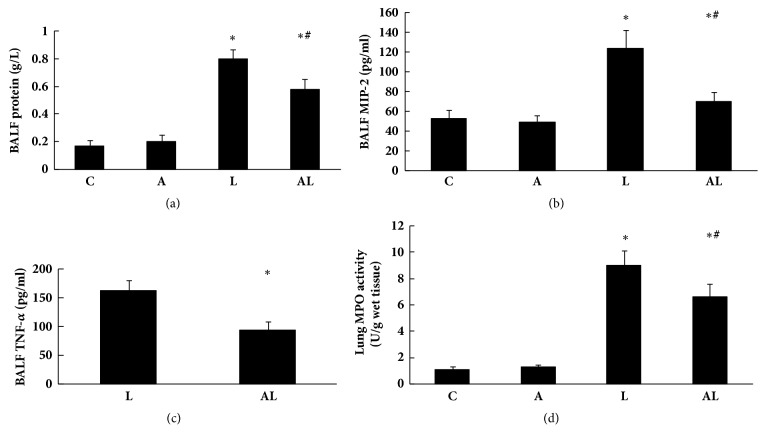
Protein, cytokines in BALF and MPO activity in lung tissue. (a) Concentrations of total protein in BALF of groups. (b) Concentrations of MIP-2 in BALF of groups. (c) Concentrations of TNF-*α* in BALF of groups. (d) Lung tissue MPO activity in groups. Values are mean ± SD (n=8 per group). ^∗^*p*<0.01 versus C group; ^#^*p*<0.01 versus L group.

Concentration of total protein in BALF of the L group increased significantly compared with the C group (*p*<0.01, Figure 3(a)). This protein concentration was significantly reduced in the AL group (*p*<0.01). However, it was significantly higher than that of the C group (*p*<0.01). Concentrations of MIP-2 in BALF of each group were similar to total protein levels (Figure 3(b)). TNF-*α* in BALF of the C group and the A group could not be detected. Concentration of TNF-*α* in BALF of the AL group was significantly lower than that of the L group (*p*<0.01, Figure 3(c)).

Similarly, lung tissue MPO activity in the L group increased significantly compared with the C group (*p*<0.01, Figure 3(d)). This increase in lung tissue MPO activity was significantly reduced in the AL group (*p*<0.01). However, it was significantly higher than that of the C group (*p*<0.01).

### 3.4. Expression of NHE-1, MAPK p38, p- p38, ERK, and p-ERK in Lung Tissue

**Figure 4:**
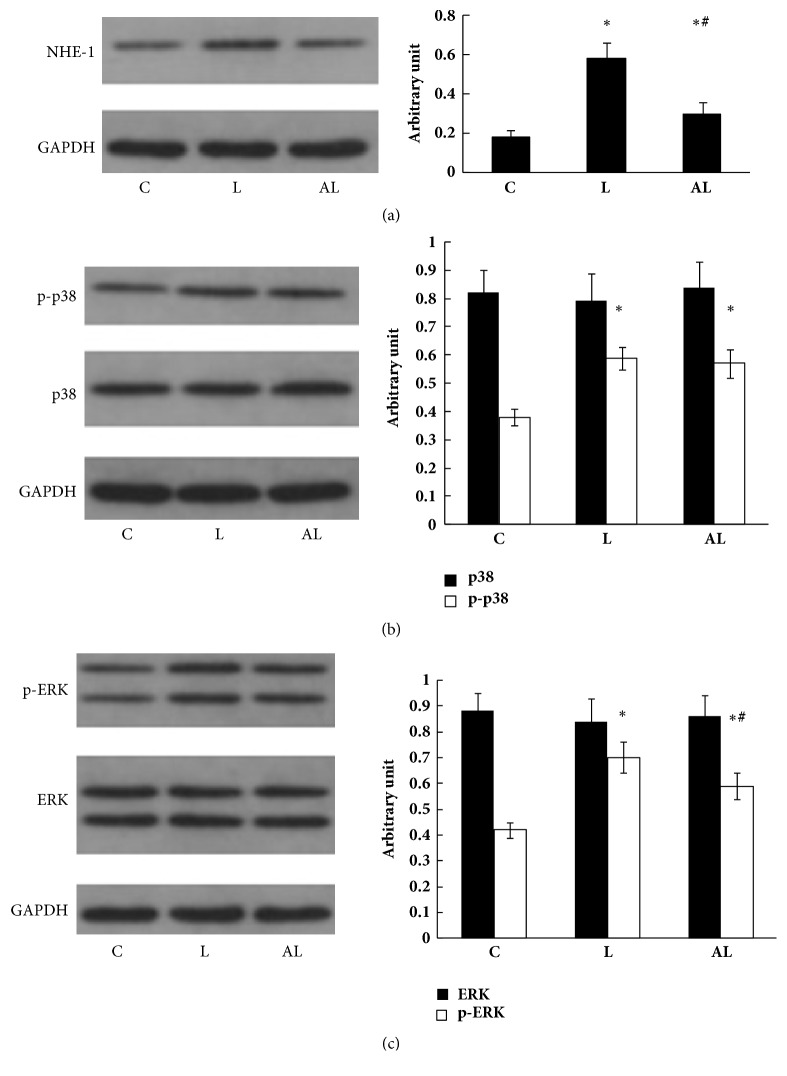
Western blot analysis of NHE-1 (a), p38 and p-p38 (b), ERK and p-ERK (c) in lung tissue. Lane (C) C group; Lane (L) L group; Lane (AL) AL group. The left panel shows western blot of proteins in lung tissue. The right panel shows relative densitometric units. Data are expressed as mean ± SD of five independent experiments. ^∗^*p*<0.01 versus C group; ^#^*p*<0.01 versus L group.

Expression of NHE-1 in the L group increased significantly compared with the C group. Increased NHE-1 expression was significantly reduced in the AL group. However, it was higher than that of the C group (Figure 4(a)). Expression of p-p38 and p-ERK in the L group increased significantly compared with the C group. Increased p-ERK expression was partly reversed in the AL group, whereas increased p-p38 expression was not (Figures 4(b) and 4(c)).

## 4. Discussion

In the present study, intravenous administration of LPS caused ALI in rats, as evidenced by high levels of W/D, ALI score, and MPO activity in lung tissue as well as high levels of total protein, TNF-*α*, and MIP-2 in BALF. Furthermore, these changes were associated with increased expression of NHE-1, MAPK p-p38, and p-ERK. In the case of NHE-1 inhibition by amiloride pretreatment, ALI was significantly inhibited, and this was associated with decreased NHE-1 and p-ERK expression. These results indicate that NHE-1 plays an important role in the development of inflammation in ALI. NHE-1 inhibition by amiloride exerts potent anti-inflammatory effect in rat lung following ALI and this may be associated with reversal of ERK.

Neutrophils play a key role in the pathogenesis of ALI, causing cell damage through the production of free radicals, inflammatory mediators, and proteases [20]. They are the dominant leukocyte type found both in BALF and in histological specimens from patients with ARDS [21]. In rats with ALI in the L group, MPO activity, an indicator of neutrophil recruitment into lung tissue, was significantly increased. The recruitment of neutrophils to the lung in response to LPS-induced injury may be dependent on the coordinated expression of proinflammatory cytokines, adhesion molecules, and the establishment of chemotactic gradients via the local generation of chemotactic factors. There is accumulating evidence that NHEs are involved in the regulation of this process. NHE-1 inhibition by amiloride inhibits TNF-*α* production in LPS-stimulated human alveolar macrophages [10]. TNF-*α*, an early proinflammatory cytokine, is an important mediator for sepsis and concomitant lung injury. In conjunction with IL-1*β*, TNF-*α* activates the inflammatory cascade by promoting the production of several cytokines and chemokines, and by enhancing endothelial adhesion molecule expression on vascular endothelial cells that promotes neutrophil adherence to these cells [2]. NHEs have been implicated in the regulation of chemokine production. NHE-1 inhibition by amiloride suppresses IL-8 production in respiratory epithelium infected with respiratory syncytial virus, LPS-stimulated human alveolar macrophages, and LPS-stimulated endothelial cells [10, 11, 22]. NHE-1 inhibition by amiloride also suppresses MIP-2 production by LPS-stimulated mouse macrophages [14]. MIP-2 is a rodent homologue of human IL-8, and both are important chemokines of neutrophil recruitment and activation. In addition, amiloride suppresses IL-1*β*-induced E-selectin expression in endothelial cells [11]. Furthermore, volume changes in neutrophils caused by NHE activation facilitate migration in vitro [23]. These observations suggest that NHE activation may facilitate neutrophil activation, adhesion, and transmigration. In the present study, pretreatment of rats with NHE-1 inhibitor amiloride attenuated the pulmonary neutrophil infiltration, TNF-*α*, and MIP-2 production by inflammatory cells, resulting in attenuation of ALI induced by LPS. Consistent with our findings, coadministration of amiloride and hypertonic saline was more efficacious than administration of hypertonic saline alone in reducing trauma-hemorrhagic shock-induced pulmonary permeability and neutrophil sequestration and further protected lungs [24].

There is accumulating evidence that the activation of NHEs contributes to endothelial cell injury during many pathophysiological processes associated with acidosis and hypoxia [15]. Inhibition of NHEs prevents the increase in lung endothelial permeability induced by either septic shock or hemorrhagic shock. Acidosis and hypoxia are implicated in ALI induced by LPS. In the present study, pretreatment of rats with NHE-1 inhibitor amiloride attenuated pulmonary permeability. W/D and total protein in BALF of the AL group were significantly decreased.

NHE-1 is ubiquitous in its distribution, including expression in the lungs. Studies suggest that an increase in intracellular O_2_^−^ anion induces NHE-1 gene promoter activity resulting in increased NHE-1 protein expression [25]. Neutrophils accumulated in the lung during ALI release free radicals, including O_2_^−^ anion, causing an increase in intracellular O_2_^−^ anion. Elevation of intracellular O_2_^−^ anion induces NHE-1 protein expression. In the present study, expression of NHE-1 protein significantly increased in the lungs of rats with ALI. NHE-1 is rapidly activated in response to a variety of inflammatory signals, such as IL-1, TNF-*α*, IFN-gamma, and LPS. Increased activation of NHE-1 induces more accumulation of neutrophils into the lung in turn. This positive feedback could amplify the inflammatory response and form a cascade, which leads to more severe damage to the lungs. Pretreatment of rats with NHE-1 inhibitor amiloride attenuated the pulmonary neutrophil infiltration; therefore the increase in NHE-1 protein expression was significantly reduced in the AL group.

NHE-1 inhibitor amiloride suppresses the production of many important cytokines, as we noted above. Since both ERK and p38 kinases are necessary for cytokine gene transcription during ALI induced by LPS [26], we further investigated the expression of p38 and ERK in lungs of rats with ALI and the effect of pretreatment with amiloride on their expression. Our findings demonstrated that expression of both p-ERK and p-p38 significantly increased in lungs of rats with ALI. Pretreatment with amiloride significantly reduced the increase in p-ERK expression but not the increase in p-p38 expression. Therefore, amelioration of LPS-induced ALI by NHE-1 inhibitor amiloride is mediated through ERK, but not through p38.

## 5. Conclusions

Activation of NHE-1 plays an important role in cytokine production and the accumulation of neutrophils in the lung in LPS-induced ALI. Pretreatment of rats with NHE-1 inhibitor amiloride can significantly reduce cytokine production and the pulmonary neutrophil infiltration, therefore attenuating ALI. This effect is mediated through ERK, but not through p38.

## Data Availability

The data used to support the findings of this study are available from the corresponding author upon request.

## References

[1] Yu J., Shi J., Wang D. (2016). Heme oxygenase-1/carbon monoxide-regulated mitochondrial dynamic equilibrium contributes to the attenuation of endotoxin-induced acute lung injury in rats and in lipopolysaccharide-activated macrophages. *Anesthesiology*.

[2] Thompson B. T., Chambers R. C., Liu K. D. (2017). Acute respiratory distress syndrome. *The New England Journal of Medicine*.

[3] Orlowski J., Grinstein S. (2007). Emerging roles of alkali cation/proton exchangers in organellar homeostasis. *Current Opinion in Cell Biology*.

[4] Kondapalli K. C., Prasad H., Rao R. (2014). An inside job: how endosomal Na(+)/H(+) exchangers link to autism and neurological disease. *Frontiers in Cellular Neuroscience*.

[5] Orlowski J., Kandasamy R. A., Shull G. E. (1992). Molecular cloning of putative members of the Na/H exchanger gene family. cDNA cloning, deduced amino acid sequence, and mRNA tissue expression of the rat Na/H exchanger NHE-1 and two structurally related proteins. *The Journal of Biological Chemistry*.

[6] Zhao Y., Cui G., Zhang N., Liu Z., Sun W., Peng Q. (2012). Lipopolysaccharide induces endothelial cell apoptosis via activation of Na(+)/ H(+) exchanger 1 and calpain-dependent degradation of Bcl-2. *Biochemical and Biophysical Research Communications*.

[7] Tattersall A. L., Browning J. A., Wilkins R. J. (2005). Modulation of H+ transport mechanisms by interleukin-1 in isolated bovine articularchondrocyts. *Cell Physiol Biochem*.

[8] Xu H., Li Q., Zhao Y., Li J., Ghishan F. K. (2016). Intestinal NHE8 is highly expressed in goblet cells and its expression is subject to TNF-*α* regulation. *American Journal of Physiology-Gastrointestinal and Liver Physiology*.

[9] Amin M. R., Dudeja P. K., Ramaswamy K., Malakooti J. (2007). Involvement of Sp1 and Sp3 in differential regulation of human NHE3 promoter activity by sodium butyrate and IFN-*γ*/TNF-*α*. *American Journal of Physiology-Gastrointestinal and Liver Physiology*.

[10] Rolfe M. W., Kunkel S. L., Rowens B., Standiford T. J., Cragoe E. J., Strieter R. M. (1992). Suppression of human alveolar macrophage-derived cytokines by amiloride. *American Journal of Respiratory Cell and Molecular Biology*.

[11] Németh Z. H., Deitch E. A., Lu Q., Szabó C., Haskó G. (2002). NHE blockade inhibits chemokine production and NF-*κ*B activation in immunostimulated endothelial cells. *American Journal of Physiology-Cell Physiology*.

[12] Németh Z. H., Deitch E. A., Szabó C. (2002). Na+/H+ exchanger blockade inhibits enterocyte inflammatory response and protects against colitis. *American Journal of Physiology-Gastrointestinal and Liver Physiology*.

[13] Kamachi F., Hyun S. B., Hirasawa N., Ohuchi K. (2007). Inhibition of lipopolysaccharide-induced prostaglandin E2 production and inflammation by the Na+/H+ exchanger inhibitors. *The Journal of Pharmacology and Experimental Therapeutics*.

[14] Németh Z. H., Mabley J. G., Deitch E. A., Szabó C., Haskó G. (2001). Inhibition of the Na+/H+ antiporter suppresses IL-12 p40 production by mouse macrophages. *Biochimica et Biophysica Acta (BBA) - Molecular Cell Research*.

[15] Wu D., Kraut J. A. (2014). Role of NHE1 in the cellular dysfunction of acute metabolic acidosis. *American Journal of Nephrology*.

[16] Yang X., Bai H., Cai W. (2013). Inhibition of Na+/H+ exchanger 1 by cariporide alleviates burn-induced multiple organ injury. *Journal of Surgical Research*.

[17] Wu D., Russano K., Kouz I., Abraham W. M. (2013). NHE1 inhibition improves tissue perfusion and resuscitation outcome after severe hemorrhage. *Journal of Surgical Research*.

[18] Zhang B., Liu Z., Li Y. (2011). Antiinflammatory effects of matrine in LPS-induced acute lung injury in mice. *European Journal of Pharmaceutical Sciences*.

[19] Wu Q., Gui P., Yao S., Zhu H., Li J., Li Y. (2011). Expression of *β*-Defensin-3 in Lungs of Immunocompetent Rats with Methicillin-Resistant Staphylococcus aureus Ventilator-Associated Pneumonia. *Journal of Surgical Research*.

[20] Blázquez-Prieto J., López-Alonso I., Huidobro C., Albaiceta G. M. (2018). The Emerging Role of Neutrophils in Repair after Acute Lung Injury. *American Journal of Respiratory Cell and Molecular Biology*.

[21] Williams A. E., Chambers R. C. (2014). The mercurial nature of neutrophils: still an enigma in ARDS?. *American Journal of Physiology—Lung Cellular and Molecular Physiology*.

[22] Mastronarde J. G., Monick M. M., Gross T. J., Hunninghake G. W. (1996). Amiloride inhibits cytokine production in epithelium infected with respiratory syncytial virus. *American Journal of Physiology-Lung Cellular and Molecular Physiology*.

[23] Rosengren S., Henson P. M., Worthen G. S. (1994). Migration-associated volume changes in neutrophils facilitate the migratory process in vitro. *American Journal of Physiology*.

[24] Fujiyoshi N., Deitch E. A., Feketeova E. (2005). Amiloride combined with small-volume resuscitation with hypertonic saline is superior in ameliorating trauma-hemorrhagic shock-induced lung injury in rats to the administration of either agent alone. *Critical Care Medicine*.

[25] Akram S., Teong H. F. C., Fliegel L., Pervaiz S., Clément M. V. (2006). Reactive oxygen species-mediated regulation of the Na^+^- H^+^ exchanger 1 gene expression connects intracellular redox status with cells' sensitivity to death triggers. *Cell Death & Differentiation*.

[26] Cosin-Roger J., Spalinger M. R., Ruiz P. A. (2018). Gp96 deficiency affects TLR4 functionality and impairs ERK and p38 phosphorylation. *PLoS ONE*.

